# Induced-anxiety differentially disrupts working memory in generalized anxiety disorder

**DOI:** 10.1186/s12888-016-0748-2

**Published:** 2016-03-15

**Authors:** Katherine E. Vytal, Nicole E. Arkin, Cassie Overstreet, Lynne Lieberman, Christian Grillon

**Affiliations:** Section on Neurobiology of Fear and Anxiety, National Institute of Mental Health, 15K North Drive, MSC 2670, Bethesda, MD 20892-2670 USA

**Keywords:** Generalized anxiety disorder, Working memory, Anxiety, Startle reflex, Threat of shock

## Abstract

**Background:**

Anxiety is characterized by a bias towards threatening information, anxious apprehension, and disrupted concentration. Previous research in healthy subjects suggests that working memory (WM) is disrupted by induced anxiety, but that increased task-demand reduces anxiety and WM is preserved. However, it is unknown if patients with generalized anxiety disorder (GAD) can similarly normalize their performance on difficult WM tasks while reducing their anxiety. Increased threat-related bias and impoverished top-down control in trait anxiety suggests that patients may not reap the same cognitive and emotional benefits from demanding tasks that those low in anxiety. Here we examine this possibility using a WM task of varying difficulty.

**Methods:**

GAD patients (*N* = 30) and healthy controls (*N* = 30) performed an n-back task (no-load, 1-back, 2-back, and 3-back) while at risk for shock (threat) or safe from shock (safe). Anxiety was measured via startle reflex and self-report.

**Results:**

As predicted, healthy controls’ performance was impaired under threat during low-load tasks and facilitated during high-load tasks. In contrast, GAD patients’ performance was impaired under threat regardless of WM load. Anxiety was reduced as cognitive load increased in both groups.

**Conclusions:**

The divergence of emotion regulation (reduction) and performance (persistent impairment) in the patient but not the control group, suggests that different top-down mechanisms may be operating to reduce anxiety. Continued WM disruption in patients indicates that attentional resources are allocated to emotion regulation instead of goal-directed behavior. Implications for our understanding of cognitive disruption in patients, and related therapeutic interventions are discussed.

## Background

A growing body of research examining the impact of anxiety on working memory (WM) suggests that while adaptive anxiety (i.e., anxiety in healthy individuals) can disrupt WM [1–4], an increase in task demands may successfully direct attention away from anxiety [1, 2]. Although this latter effect has positive implications for overcoming anxiety, a key element to consider is that these studies were only conducted in healthy individuals. While models of anxiety have been relatively effective in elucidating anxiety-related changes in cognition [5], they are by no means perfect replicas of anxious pathology. Pathological anxiety is characterized by attentional bias towards threat [6]), and anxiety can alter perception very early in the processing stream [7], perhaps at the expense of other goal-directed information. Patients with generalized anxiety disorder (GAD), in particular, demonstrate a predominance of verbally-mediated worry [8], consuming critical cognitive resources. We propose that difficulty in disengaging from threat results in a fundamental hijacking of the attentional system that would normally shift to support WM processes. Moreover, this effect should be specifically evident in verbal WM tasks that may share resources with verbally-mediated worry. As a result, verbal WM performance may be disrupted in GAD patients even if a task is very engaging.

### Pathological anxiety and working memory: a gap in the literature

While it is clear that anxiety is associated with an attentional bias towards threats, there is little research actually demonstrating that *active*^1^ anxious pathology disrupts cognition. The majority of WM research in patients has been conducted in the absence of an anxiogenic stressor [9, 10] and the effects are equivocal. This lack of consistent findings contrasts with the clinical observation of a greater susceptibility to distraction in anxious patients and may be the result of the fact that cognitive impairments are studied in safe laboratory environments. To our knowledge, no study has examined anxious patients’ WM during an anxiogenic challenge.

In support of the link between anxiety and WM impairment, other work has shown that trait anxiety, a marker of vulnerability to pathology, is associated with cognitive disruption [11–15]. For example, trait anxiety is associated with executive function impairment when a visuospatial WM task and a finger taping task is performed concurrently, but not when it is performed alone [11]. Other work has shown that individuals with dispositional worry [14] and dispositional anxiety [15] experience WM disruption when confronted with threatening distractors. These findings suggest that anxiety-related WM impairment may not be evident without placing additional demand on the cognitive system (either with a dual-task or emotion induction). Additional research in GAD patients, who tend to exhibit both dispositional anxiety and dispositional worry, is needed to characterize the relationship between pathological anxiety and verbal working memory.

### Top-down control mechanisms in adaptive versus pathological anxiety

While examining the impact of anxiety on cognitive disruption is critical, it is equally important to investigate anxiety reduction mechanisms. Previous research suggests that engaging tasks may effectively reduce shock-induced anxiety in healthy participants [1] to a similar extent as active emotion regulation strategies like reappraisal [16] or detachment [17, 18]. The basic principle across these strategies is the same: top down executive control mechanisms refocus attention and indirectly (or directly) down regulate anxiety. In contrast, a separate line of research suggests that trait anxiety may actually become *more* disruptive when cognitive tasks are challenging [19, 20]. This discrepancy illustrates the difference between state and trait anxiety. The attentional control theory proposes that cognitive disruption occurs because anxiety activates a stimulus-driven network (bottom-up) at the expense of a goal-driven network (top-down control) [21]. Thus, active adaptive anxiety may engage a stimulus-driven system when task demands are low, leaving cognitive processes susceptible to disruption, while more demanding tasks preferentially engage a goal-driven system leading to successful performance in the face of threat. On the other hand, active pathological anxiety and active trait anxiety may intractably activate the stimulus-driven system, with impoverished top-down control [22, 23] leading to cognitive disruption regardless of task difficulty.

### The current study

Here we explored the impact of anxious pathology on working memory when subjects are in a threatening environment (anticipating a shock), and conversely, the impact of task difficulty on performance in the face of threat. GAD patients and healthy control participants performed a verbal n-back task of varying difficulty (view [no response], 1-back, 2-back, and 3-back) while at risk for shock and while safe from shock. The startle reflex served as a probe of anxiety [24]. We expected to replicate findings in healthy individuals by using an identical paradigm [1, 2], showing that induced-anxiety disrupts n-back WM under lower-load tasks and performance is normalized under threat on higher-load tasks. We predicted that patients with GAD would be impaired regardless of task difficulty, with active anxiety disrupting executive control mechanisms that direct attention towards a task. The possibility of persistent WM disruption under high cognitive load in anxiety patients would pinpoint a critical link between pathological anxiety and perseverative cognitive impairment, which may inform therapeutic interventions and etiologic mechanisms.

## Methods

### Participants

Sixty-two subjects received monetary compensation for participation in the study. Upon arrival at the National Institutes of Health, participants completed an intake evaluation consisting of a physical exam, urine screen, and a Structured Clinical Interview for assessing DSM-IV Axis I psychiatric diagnoses [25]. Exclusion was based on the following criteria: 1) acute or chronic medical condition (other than psychopathology), 2) past or current personal or family history of psychiatric disorder(s) (healthy only), 3) past or current cognitive behavioral therapy (CBT) treatment, 4) use of psychoactive medications in past month, or illicit drugs (e.g., marijuana, cocaine etc.) usage, 5) lifetime history of alcohol or drug dependence, 6) current pregnancy or breast-feeding. Participants who met criteria for GAD as a primary diagnosis without comorbid depression were included in the study. The most common secondary diagnosis of GAD patients (*N* = 8) was social anxiety disorder. Two participants were excluded because of incomplete data. The final group of participants consisted of 30 healthy subjects (15 male; mean age 27 years) and 30 patients with a primary diagnosis of GAD (13 male; mean age 31 years) (see Table 1 for a breakdown of group demographics). A sample of 30 participants in each group was selected based on previous WM research that has had sufficient power to detect effects using a similar paradigm [1, 2], as well as cognitive research that has identified between-group effects using GAD patient samples [26, 27]. Wechsler Abbreviated Scale of Intelligence (WASI; [28]) scores did not differ between groups. The study design and consent documents were approved by the Combined Neuroscience Institutional Review Board of the National Institutes of Health. After the procedures were fully explained, all subjects provided written informed consent.

**Table 1:** Sample demographics

Group	N (M/F)	Age			WASI		
		*M*	*SD*	*t*	*M*	*SD*	*t*
GAD patients	30 (13/17)	31	9.5	1.7	119.5	12.3	0.2
Healthy controls	30 (15/15)	27	7.3		119.0	10.4	

M = number of males, F = number of females. All *t*-scores NS

### Paradigm

**Fig. 1:**
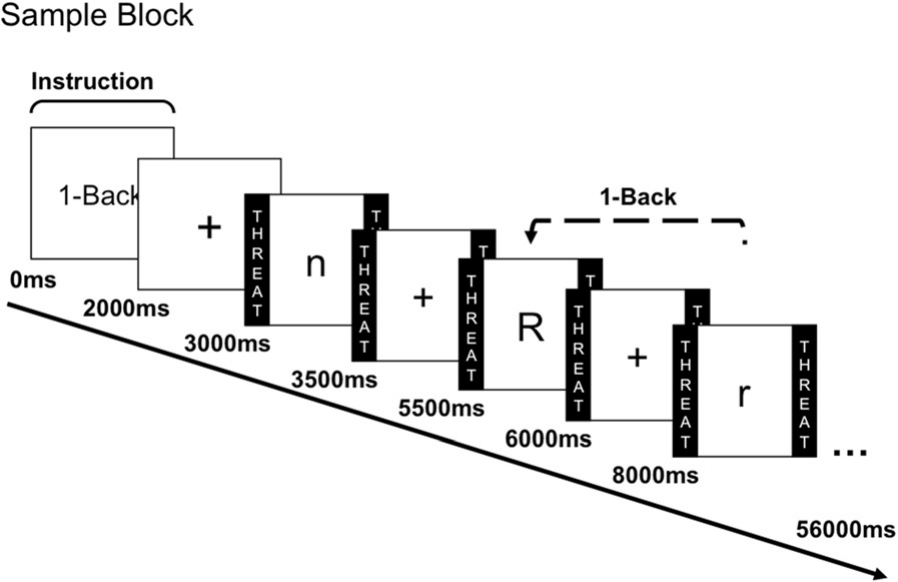
Sample Block. Paradigm consisted of alternating period of threat and safe, indicated by colored borders with the same respective labels. Each task block began with an instruction screen (view, 1-back, 2-back, or 3-back), followed by a fixation cross. Letters were presented for .5 s, and separated by a 2 s ITI. Participants responded to each letter based on whether the letter was the same as the letter one, two, or three trials back, depending on the task

The paradigm used was identical to that of [1]. Briefly, subjects engaged in a verbal n-back task of varying difficulty (view, 1-back, 2-back, and 3-back) during period of threat (risk for shock to the wrist; 0-2 shocks were delivered in each block) and safety (no shock) (see Fig. 1 for a sample block). All subjects had experience with the shock prior to the experiment during a shock work-up procedure. Based on this work-up, the shock was individually set at a level that was uncomfortable, but not painful for each participant. Before each 50-s task block, an instruction screen indicated the task to be performed. Blocks alternated between threat and safe, and were separated by an 8 s ITI. Participants responded to each letter based on whether the letter was the same or different than one, two, or three trials back (n-back load order was randomized). In the view condition, subjects simply attended to the stimuli without making a button press. Startle was probed with a short blast of white noise every 18-22 s, in both threat and safe conditions. Following each block of stimuli, subjects made a retrospective anxiety rating that assessed their subjective anxiety during the previous block on a scale from 1 (little to no anxiety) to 9 (high or extreme anxiety).

### Equipment

Presentation software (neurobehavioral systems) was used to present visual stimuli on the computer screen and control the presentation of shocks and startle probes. Contact Precision Instruments equipment was used to present shock stimuli and record facial EMG responses. A Digitimer constant current stimulator (DS7A; Digitimer, UK) was used to present shocks. Shocks were administered using two 6 mm Ag/AgCl electrodes that were attached to the median nerve of the left wrist.

### Data reduction and analysis

EMG data were sampled at 1000 Hz, filtered (30-500Hz), rectified, and smoothed with a 20-ms time constant. Startle responses were defined as the peak magnitude of the eye blink reflex (20–100 ms after stimulus onset) relative to a 50-ms average baseline that immediately preceded the probe onset. Baseline artifacts resulted in the exclusion of less than one percent of trials. Peak eye blink magnitudes were T-scored (across all conditions) and averaged within each condition for each subject to attenuate large inter-individual differences in raw reflex magnitude. To confirm that T-score transformation did not introduce any artifact that might alter our effects, we conducted all startle analyses with both the raw startle data and the transformed data. The effects did not differ between datasets, and therefore we only report the findings based on T-scored data. Psychometric equivalency for this paradigm was determined in previous studies [1, 2], demonstrating that any potential anxiety-related performance differences in low load versus high load tasks cannot be attributed to greater discriminating power (see [3] for a detailed discussion of the importance of psychometric analysis in the absence of a double dissociation). To confirm that performance accuracy did not differ as a result of shock or probe administration, trials that preceded or followed shocks, and those that preceded or followed probes were analyzed separately. No differences were found and all trials were included in the final analysis. To address the possibility of speed-accuracy tradeoffs, we analyzed RT data using repeated-measures ANOVAs both within (Anxiety x Load) and between subject groups (Group x Anxiety x Load). Omissions were uncommon and unsystematic in the n-back task; however, we excluded trials where participants failed to respond before the next stimulus appeared on the screen (i.e., 2500 ms post-stimulus onset). All participants performed above chance. Anxiety ratings and startle potentiation were examined using 2 (Group: patient, control) x 2 (Anxiety: threat, safe) x 4 (Load: view, 1-back, 2-back, 3-back) repeated-measures ANOVAs. Performance differences in accuracy were examined using a 2 (Group: patient, control) x 2 (Anxiety: threat, safe) x 3 (Load: view, 1-back, 2-back, 3-back) repeated-measures ANOVA. Greenhouse-Geisser corrections (GG-ε) were used for repeated-measures ANOVAs that involved factors with three or more levels. In addition to this guard against sphericity violations, assumptions of normality for paired sample *t*-tests (Shapiro-Wilk’s *W* test) and equality of variances for repeated-measures ANOVAs (Levene’s Test for Equality of Variances) were assessed and met. Alpha was set at 0.05 for all statistical tests. All data were analyzed using the Statistical Package for Social Sciences (SPSS).

## Results

### Manipulation checks

**Fig. 2:**
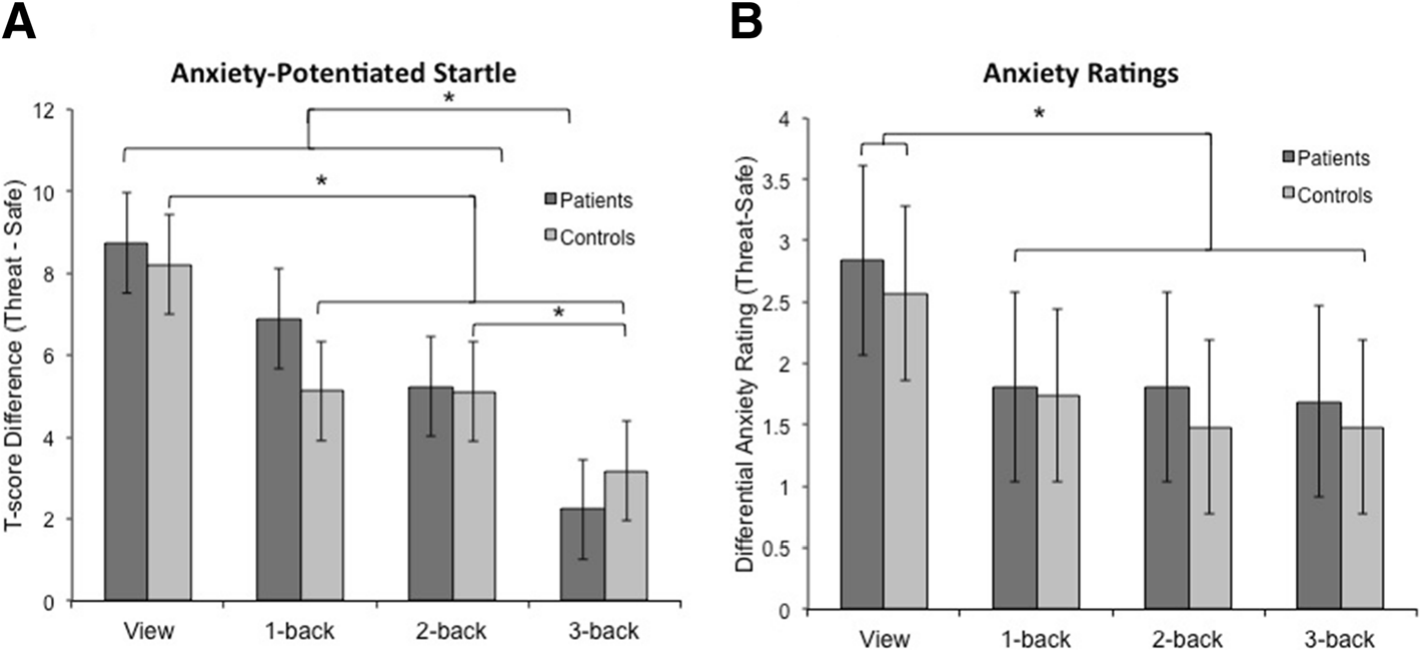
Anxiety-potentiated startle and differential anxiety ratings across load. **a** GAD patient and healthy control anxiety-potentiated startle (APS; threat-safe) was consistently elevated, suggesting anxiety was higher in threat versus safe conditions. APS was reduced as task difficulty increased, indicating that anxiety was regulated with an increase in load. Paired sample *t*-tests demonstrated that patients’ APS during 3-back was significantly lower than other conditions. Healthy controls’ APS during view was significantly higher than all other conditions, and APS during 3-back was also lower than 2-back. **b** Anxiety ratings for both groups were lower when subjects were engaged in the working memory task versus when they were simply viewing the stimuli. Paired-sample *t-*tests indicate that ratings during view were significantly higher than all other levels of load. Asterisk indicates *p* < .05

The main effect of Anxiety (*F*(1,59) = 127.8, *p* < .001), demonstrated that subjects reported experiencing more anxiety when they were at risk for shock than when they were safe (GAD patients: threat *M* = 4.5, safe *M* = 2.5, *t*(29) = 8.6, *p* < .001; controls: threat *M* = 3.5, safe *M* = 1.7, *t(*29) = 7.5, *p* < .001). Anxiety ratings were reduced during threat versus safe conditions as WM load increased (Anxiety x Load interaction) (*F*(1,59) = 127.8, *p* < .001, *η*^*2*^ = .69), but there was no Group x Anxiety x Load interaction (*F*(1,59) = .4, *p* = .521, *η*^*2*^ = .01), indicating patients and controls were similarly anxious in the threat versus safe condition as WM load increased (see Fig. 2b for anxiety ratings across load). Furthermore, patients were not significantly more anxious than controls in the threat versus safe condition (*t*(58) = .65, *p* = .521, GAD threat-safe rating difference *M* = 2.0, *SD* = 1.3; control threat-safe rating difference: *M* = 1.8, *SD* = 1.3).

In addition to self-report, we used startle magnitude to verify that threat of shock successfully induced anxiety. Startle magnitude did not differ between patients and controls during threat versus safety across WM load (Group x Anxiety x Load repeated-measures ANOVA: *F*(3,174) = .7, *p* = .524, *η*^*2*^ = .16) (see Table 2 for *Ms* and *SD*s, and Fig. 2a for anxiety-potentiated startle across load). Similarly, startle magnitude did not differ between patients and controls during threat versus safety (Group x Anxiety repeated measures ANOVA: *F*(1,58) = .09, *p* = .772, *η*^*2*^ = .00). Nevertheless, as predicted, the main effect of Anxiety demonstrated that startle was consistently potentiated by threat of shock, *F*(1,59) = 80.2, *p* < .0001, *η*^*2*^ = 1.00, confirming the manipulation. Moreover, anxiety-potentiated startle (threat – safe) was reduced by load (*F*(3,177) = 13.4, *p* < .001, *η*^*2*^ = 1.0), indicating that load decreased anxiety (confirmed by a linear trend: *F*(1,59) = 10.5, *p* < .005, *η*^*2*^ = .89). Paired-sample *t*-tests showed that view anxiety-potentiated startle was significantly higher than all other levels of load (1-back: *t*(58) = 2.4, *p* < .05; 2-back: *t*(58) = 2.7, *p* < .05; 3-back *t*(58) = 5.0, *p* < .001), and 3-back anxiety potentiated startle was significantly lower than all levels of load (1-back: *t*(58) = 4.1, *p* < .001; 2-back: *t*(58) = 3.5, *p* < .005).

**Table 2:** Startle T-scores across subject groups

	Threat				Safe			
Group	View	1-Back	2-Back	3-Back	View	1-Back	2-Back	3-Back
Startle potentiation								
GAD patients	55.9(7.1)	52.1(6.1)	51.3(5.5)	50.3(5.9)	47.2(10.8)	45.2(4.9)	46.0(5.7)	48.0(5.0)
Healthy controls	56.2(5.3)	51.7(3.5)	52.5(3.4)	51.3(4.2)	48.0(4.5)	46.6(3.1)	47.4(2.8)	48.1(3.0)

All startle values are listed in T-score units. Standard deviations listed in parentheses

### Performance

**Fig. 3:**
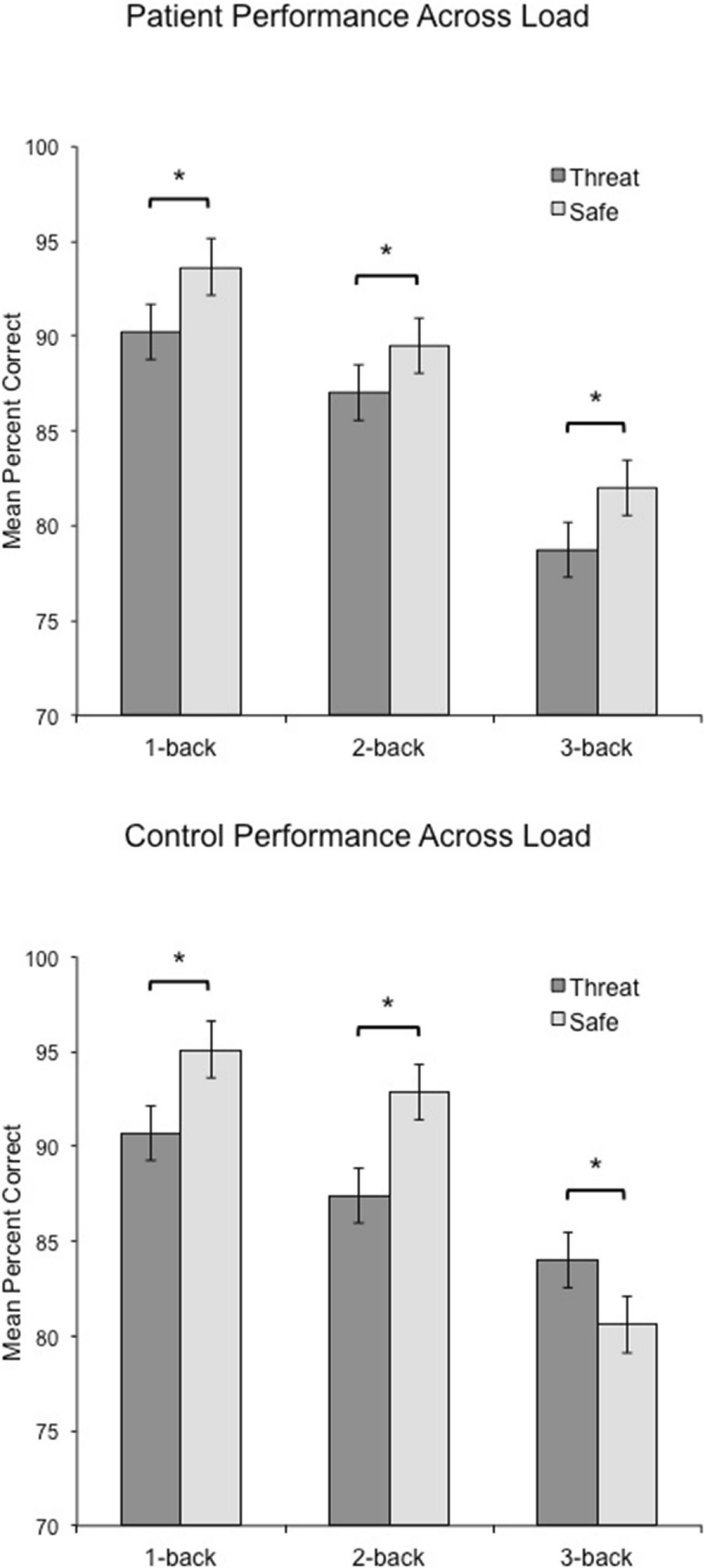
Threat and safe performance across load in patients and healthy controls. GAD patient and healthy control performance was impaired in 1-back and 2-back tasks during threat versus safe conditions. However, in 3-back, patient performance was still impaired while control performance was facilitated during threat. Asterisk indicates *p* < .05

Consistent with our predictions, the three-way interaction between Group (patient, control), Anxiety (threat, safe), and Load (1-back, 2-back, 3-back), was significant (repeated measures ANOVA, *F*(2,118) = 58, *p* = .001, *η*^*2*^ = .21), indicating that GAD patients and healthy controls exhibited a different profile of anxiety-related WM impairment in accuracy across load (see Fig. 3 for patient and control performance). The interaction between Group and Anxiety at 3-back was significant (*F*(1,58) = 19.2, *η*^*2*^ = .25), demonstrating that induced anxiety has a different effect on GAD patient 3-back performance versus healthy control 3-back performance. To further decompose this interaction, performance data were analyzed separately for patients and healthy controls .

The Anxiety and Load interaction for WM performance in GAD patients was not significant, *F*(2,60) = .3, *p* = .770, *η*^*2*^ = .01. However, there was a significant main effect of Anxiety on performance, *F*(1,30) = 27.3, *p* < .001, *η*^*2*^ = .476, indicating that WM performance was impaired overall during threat as compared to safe, regardless of task difficulty (see Fig. 2). This finding indicates that under both low and high cognitive load, an anxiogenic context impaired WM in patients (threat – safe 1-back, *t*(29) = -4.0, *p* < .01; 2-back, *t*(29) = -2.4, *p* < .05; and 3-back, *t(*29) = -3.3, *p* < .005).

In contrast to patients, healthy controls showed a significant interaction of Anxiety and Load, *F*(2,58) = 18.7, *p* < .001, *η*^*2*^ = .39, reflecting the finding that 1-back and 2-back performance accuracy was impaired during threat as compared to safe (*t*(29) = -4.1, *p* < .001, and *t*(29) = -4.7, *p* < .001, respectively), but 3-back performance was facilitated by threat (*t*(29) = 2.9, *p* = .006) (see Fig. 3). These findings suggest that in healthy controls, lower-demand tasks are susceptible to disruption by induced-anxiety, whereas higher-demand tasks are facilitated by an adaptive anxiety. A comparison of patient and healthy control accuracy during the safe condition suggests a trend toward performance differences, but did not reveal a significant effect *F*(1,57) = 3.0, *p* = .06). Further investigation of performance during the safe condition, confirmed similar performance between groups (*t*(58) = .706, *p* = .483), suggesting that performance in the absence of an anxiogenic challenge was not driving force of these group effects.

Omnibus tests were conducted for the main effects of Group and Load on performance. The main effect of Group was not significant, suggesting no overall performance differences between groups when all factors were collapsed (*F*(1,58) = .84, *p* = .364). As expected, the main effect of Load was significant, indicating that WM difficulty affected performance (*F*(2,58) = 18.7, *p* < .001, *η*^*2*^ = .39). *T*-tests confirmed that performance significantly decreased from 1-back to 2-back, and 2-back to 3-back (*t*(59) = 4.5, *p* < .001; 2-back, *t*(59) = 12.3, *p* < .001, respectively). Thus, performance decreased as task difficulty increased.

### Reaction time

For both the patient and control groups, we confirmed that RT did not differ between threat and safe across Load, (patient: *F*(2,56) = 1.4, *p* = .249; control: *F*(2,56) = .0, *p* = .981). More specifically, RT differences (threat-safe) were not significantly different for any of the tasks (patients: controls: threat – safe 1-back, *t*(29) = -1.2, *p* = .245; and 2-back, *t*(29) = -.0, *p* = .964, controls: threat – safe 1-back, *t*(29) = .3, *p* = .734; 2-back, *t*(29) = .3, *p* = .800; and 3-back, *t*(29) = .2, *p* = .858), except for 3-back in the patient group, *t*(29) = 2.4, *p* = .021. However, this significant effect was in the same direction as the performance data (i.e., performance accuracy was lower and RT was higher during 3-back threat), indicating that there was not a speed-accuracy tradeoff.

To address the possibility of speed accuracy tradeoffs between subject groups, we conducted a repeated measures ANOVA on the reaction time (RT) data between groups, which demonstrated no significant interaction among Group, Condition, and Load, *F*(2, 116) = 1.5, *p* = .231, between Group and Condition (*F*(2, 58) = 1.7, *p* = .205), and between Group and Load (*F*(2, 116) = .4, *p* = .640). These findings indicate that the differential performance of patients and healthy controls was not driven by differences in response time (i.e., speed-accuracy tradeoffs).

## Discussion

Despite the fact that anxious patients complain of being highly distractible by their anxiety, the present report found no impairment in performance in the safe condition. This is consistent with many reports reporting normal performance on a range of cognitive tasks in anxiety disorders [9, 29, 30]. However, our results showed performance impairment in the GAD group during an anxiogenic challenge. Specifically, the GAD patients exhibited continued disruption of WM regardless of task difficulty during threat of shock. Although the healthy controls showed impaired performance during threat under low WM load and facilitated performance during threat under high WM load, anxious patients did not exhibit cognitive benefits from engaging in more difficult tasks when anticipating shocks. In contrast, overall levels of anxiety did not differ between patients and controls, as indicated by startle and behavioral ratings. These findings indicate that anxiety regulation (indirect or direct) related to task demand is effective in both groups. However, the performance difference at 3-back indicates that GAD patients’ focus on threat was not ameliorated by cognitive load. Together, these findings suggest that top-down control of anxiety may occur through qualitatively or quantitatively different mechanisms in healthy controls and anxiety patients. Here, we suggest that explicit top-down control of anxiety, rather than anxiety itself, interferes with GAD WM performance under high WM load.

### Top-down control of anxiety: a different mechanism in anxious pathology?

On the surface it would appear that WM load reduces perceived anxiety and defensive responding in similar ways for both GAD patients and healthy controls: startle magnitude and anxiety ratings in threat versus safe are reduced as load increases. Yet our WM data suggest that the effects of anxiety are still present in GAD patients. If WM performance is an indicator of anxious disruption, as our findings suggest, then while perceived anxiety and defensive responding decreases, the deleterious effects of anxious pathology persist under difficult tasks. Why might this occur? First, the disruption might result from impoverished prefrontal control mechanisms in pathological anxiety. Previous research suggests that trait anxiety, a potential marker for pathology, is associated with poor top-down cognitive control [22, 31]. However, our patient group showed intact regulation of anxiety as measured by self-report and startle, with the rate of reduction statistically identical to our control group. Impoverished control may result in disrupted cognition but it should also be associated with preserved anxiety, not effective reduction of anxiety. Further, in trait anxiety, this effect was observed only under easy tasks, where behavioral disruption was detected (slower reaction times) [22], indicating that this mechanism of disruption may be specific to low-load tasks.

We suggest that rather than impoverished attentional control, our findings indicate that deliberate reduction of anxiety in anxious patients may result from the activation of a different mechanism than that used by controls. Specifically, we propose that the control group experienced incidental anxiety reduction via redirection of attention to focus on a task. Studies of attentional systems suggest that healthy individuals engage two separate networks in the brain depending on whether the information being processed is task-related or stimulus-related [32]. These two competing networks appear to be somewhat mutually exclusive because when a goal or stimulus initiates activity in one network, the other becomes disengaged. Thus, rather than engaging explicit emotion regulation mechanisms, the control group may have simply switched attentional systems from shock to task as the task became more demanding and additional value was placed on performance. Furthermore, the additive effects of task stress and anxiogenic context resulted not only in improved performance, but in better performance during threat versus safety. Although not initially predicted, these results are in line with research that links acute stress with cognitive enhancements [33], and in particular WM improvements [34].

The divergence between performance and startle data may lend additional support to the claim that GAD patients are engaging a different cognitive mechanism than controls. A significant interaction among Group, Anxiety, and Load indicates that the profile of anxiety-related performance deficits differed across groups as task difficulty increased. However, this same interaction was not present in the startle data. Specifically, while both groups demonstrated a reduction in anxiety-potentiated startle as WM load increased, GAD patients showed impaired performance across load, and healthy controls showed impaired performance under low load and improved performance under high load. In contrast to the control group, which appears to regulate anxiety by focusing on the task, we find that the patients regulated their anxiety (evidenced by a reduction in anxiety-potentiated startle) without improving their performance in the high load condition. During low load, the performance of all participants is disrupted by anxiety induction because threat of shock is more salient than the task (i.e., when task-related cognitive load is low). However, when the task is more engaging, we propose that healthy controls switch from a threat-driven to a task-driven attentional system (reducing their anxiety and increasing their performance) while GAD patients continue to explicitly focus on threat and threat-reduction (reducing their anxiety and reducing their performance). This dissociation suggests that GAD patients may have preferentially focused on emotion down-regulation as opposed to task performance.

Ochsner and Gross [35] propose a continuum of cognitive control of emotion on which task-related distraction and appraisal/reappraisal lie on opposite ends. Task-related distraction depends on a shift in attention from a negative emotion to a task, whereas reappraisal of a negative stimulus depends on a shift in cognition (but attention remains on the emotional stimulus). Given that GAD patients are chronically anxious, it is not surprising that these patients may have been preferentially motivated to calm their anxiety versus perform a task. We suggest that GAD patients intentionally engaged top-down regulation mechanisms (e.g., reappraisal) to control their mounting anxiety, thus resulting in the allocation of executive resources toward emotion regulation at the expense of goal-directed behavior.

This proposed qualitative disparity in anxiety reduction mechanisms between patients and controls may hinge on the conscious activation of top-down regulation mechanisms (patients) versus attentional redirection (healthy controls). Indeed, when trait anxious subjects are presented with subliminal (masked) distractors, there is no effect on cognition, but supraliminal distractors readily disrupt cognition in trait anxious subjects [31]. These findings suggest that conscious top-down control may be particularly subject to interference in anxious pathology. This hypothesis should be explicitly explored by using the current study’s paradigm in the context of a neuroimaging experiment to identify neural mechanisms of regulation and disruption in pathological anxiety. By comparing the connectivity of brain regions during threat versus safe across load, we can test the claim of whether or not patients and controls engage in different regulatory mechanisms.

### Strengths and limitations

Among the strengths of the present study are the fact that we used an established anxiety induction method, we investigated adaptive and pathological anxiety by examining GAD patients and healthy controls, and we used a paradigm that allowed us to parametrically modulate WM load. By using a robust translational method of anxiety induction like threat of shock that can be turned on and off in the same experimental run, each subject served as his or her own control. We were thus able to make inferences about pathology, and more specifically about active pathology (i.e., when an anxiety patient is in an anxious state), as well as adaptive anxiety. Further, the use of translational research methods facilitates comparison between human and animal research, thus providing an important informational bridge across domains.

Although a within-subject paradigm was used to detect group differences, 1) we cannot, with absolute certainty, attribute differences to the presence or absence of anxious pathology in isolation from potential comorbid pathology 2) we cannot make conclusions about the effect of anxiety on other types of WM. However, given that we induced an anxious state, it is much less likely that our findings were the result of a manifestation of pathology unrelated to anxiety. Additional research in other WM domains (e.g., spatial) will need to be conducted to make domain general and domain specific conclusions regarding the impact of adaptive and maladaptive anxiety on WM. Our work comparing spatial and verbal WM performance during induced anxiety [2], suggests that there may be group differences in spatial WM performance as well. On the other hand, GAD verbal WM may be more affected than spatial WM. A similar paradigm could be used in patients to address this gap in the literature.

## Conclusion

Our findings suggest that GAD patients have trouble simultaneously disengaging from threat and engaging in challenging WM processes, implicating that top-down control processes prioritize anxiety reduction over task performance. Although the induction of anxiety in both patients and healthy controls disrupts WM, only healthy controls are able to abolish this disruption by engaging in a difficult task. Instead of normalizing their WM performance under threat, GAD patients continue to exhibit cognitive impairment under high WM load. These findings indicate that, unlike in adaptive anxiety, cognitive disruption in clinical anxiety is robust and persistent. This effect should be taken into consideration when cognitive strategies, like CBT, are used as a therapeutic intervention. Explicit cognitive strategies should be expanded to include attentional control in addition to emotion regulation techniques to optimize overall functioning.

## Abbreviations

APS: Anxiety-potentiated startle
GAD: Generalized anxiety disorder
WM: Working memory

## Footnotes

1 Here, we will use *active* to describe an anxious state during an anxiogenic challenge. We prefer this term over *state anxiety* because the latter conveys the idea that this type of anxiety only differs in quantity, whereas we intend to convey the idea that active anxiety may be both qualitatively and quantitatively different in patients than healthy controls. In other words, active anxiety in a patient with GAD may include excessive rumination and increased heart-rate variability, whereas active anxiety in a healthy control may only manifest itself in increased vigilance.
